# Survey of a Psychogeriatric Ward

**Published:** 1962

**Authors:** D. M. D. White

**Affiliations:** Senior House Officer, Glenside and Barrow Hospitals


### A SURVEY OF A PSYCHOGERIATRIC WARD

BY

D. M. D. WHITE, M.B., CH.B.

*Senior House Officer, Glenside and Barrow Hospitals*

On entering psychiatry from general hospital posts, I found that part of my work lay in the wards for feeble, elderly patients. I felt it might be of interest to study these patients, and to see if they had all been admitted as young psychotics and grown old in hospital, or whether they had been admitted as elderly people. Also I wondered how many of them really needed mental hospital care, and what prevented the discharge of those who did not.

Barrow is a modern hospital of some five hundred beds, and has an active policy of treatment and discharge. The 120 patients (50 males, 70 females) whom I studied occupied the "Sick Hospital", wards originally designed for the reception of those patients who became affected with some intercurrent physical illness during their stay. However, the beds had gradually become filled with elderly, feeble, but for the most part not frankly ill patients. For some time virtually all physical illness arising in younger patients had been dealt with in the other wards of the hospital, and the "Sick Hospital" had long since ceased to fulfil its original function. It had become in effect a psychogeriatric unit.

### THE FINDINGS

Amongst the patients studied, only a few had entered the hospital as young people and grown old within its walls. The majority had been elderly on admission, for which the commonest cause had been depressive illness. Table I shows how length of stay was related to diagnosis, the schizophrenics remaining longest; in fact of the 29 per cent of patients who had been in hospital for more than twenty years the majority were schizophrenics.

TABLE I

| Diagnosis | Length of Stay | |
|---|---|---|
| | Under 6 years | Over 6 years |
| Schizophrenia .. .. | 5 (9%) | 36 (56.25%) |
| Depression .. .. .. | 34 (61%) | 20 (31.25%) |
| Organic dementia .. | 17 (30%) | 8 (12.5%) |
| Total | 56 (100%) | 64 (100%) |

An assessment of the present state of each of these patients was made by me with the collaboration of the senior nursing staff on the wards and the psychiatric social workers. This included an examination of the physical state, a note of the amount and type of nursing care and supervision required, and a record of the degree of social adjustment of the patient. This last was recorded as follows:

Complete—if the patient worked well and spontaneously, was quiet, clean, and continent, with no obvious mental symptoms.

Partial —if the patient worked under guidance, was quiet, clean and continent, but was institutionalized and mildly apathetic.

Poor —if there was no working ability, with obvious mental symptoms, withdrawal, suspicion, and incomplete cleanliness and continence.

Nil —if the patient was noisy, difficult, with frank mental symptoms and usually incontinence.

Finally, taking all these factors into account, a judgment was made as to whether the patient could be cared for in a place other than a mental hospital. The results of these decisions are given in Table II.

TABLE II

| Diagnosis | Number of Patients Suitable for | |
|---|---|---|
| | Mental hospital | Other care |
| Schizophrenia .. .. | 21 | 20 |
| Depression .. .. | 21 | 33 |
| Organic dementia .. | 22 | 3 |
| Total | 64 | 56 |

It is clear from Tables I and II that the elderly population that was studied consisted of only a minority of patients who had entered hospital as young people; the majority had been elderly on admission. Further, nearly all the patients with organic dementia may be expected to spend the rest of their lives in a mental hospital (the three noted as suitable for other care would in fact have needed to be in the "chronic" wards of a general hospital, being merely more physically than mentally ill at the time of the survey). Half the schizophrenic, and nearly two-thirds of the depressive patients were quite suited for care elsewhere.

### DISCUSSION

Since so many of the elderly patients in a mental hospital could be cared for elsewhere, the question arises: Should every effort be made to get these patients out, and if so, where are they to go?

There is not much doubt as to the feelings of the patients themselves on this. They bitterly resented any suggestion of a move, and those of us connected with the survey soon came to realize that a very real hostility was being engendered towards us; some patients, mainly depressives, suffered severe and intractable relapses at the thought of discharge, which persisted, defying all therapeutic measures, until the idea was abandoned. Really they could not be blamed for their attitude, for, in return for a very small amount of domestic work, they were getting a bed, food, warmth, and company, which though perhaps hardly congenial, was nevertheless company. They were enjoying a security which they feared would be lost if they left hospital, for many of them before admission knew only too well what loneliness was like.

Relatives too, tended to take a hostile view. It seemed that they did not want to have the care of the patients themselves, nor did they wish them to go elsewhere—perhaps to a nursing home where they might be asked to contribute towards the cost, or worse still, where the patients themselves might contribute, so diminishing the amount of some long-awaited legacy. There seems little doubt that factors like these did operate; in other cases it seemed that relatives had got so used to their nearest and dearest having "gone away" that any return of the patients would have meant an intolerable disturbance of their lives. One set of relatives, hearing that we had secured a place in a home for a fragile and sweet old lady, got in touch with the proprietor and related how some years previously, when at the height of her mental illness, she had tried to attack one of the staff. The offer of a place was withdrawn. Again, a husband, who had not visited his wife for a few years, was asked by letter to see me. He ignored the letter, and hung up the 'phone whenever the social worker tried to speak to him. Eventually he won, for we gave up trying.

The question of other accommodation is, of course, a very vexed one. A few of the patients would have been quite fit to go to a home of their own, if they had one, or had any chance of setting up one. But for the most part, it would mean looking for places either in an old peoples' home, or in "Part Three accommodation"; and these are among the rarest of commodities, especially when the person for whom the place is sought is already in hospital.

So it seems that the mental hospitals, like the general hospitals, must accept the idea of carrying a proportion of elderly people not really needing their specialized care. In saying this, I am assuming that a similar picture is to be found in other hospitals, and that the results of this survey are not peculiar to the hospital concerned, and this is, as far as I can find out, a likely assumption. It may be that the mental hospitals will have to accept the idea of carrying a substantially greater proportion of elderly people than do the general hospitals, if for no other reason than that quite a few elderly people come to the notice of psychiatrists lonely, underfed, vaguely paranoid or depressed, and have to be admitted more for social reasons than anything; and then they improve spontaneously, with very little or no active treatment.

Yet in many ways the mental hospitals seem no better equipped than the general hospitals for caring for these old people. Certainly it is so here. It seems to be a problem that has gradually arisen, and, just as in general hospitals, these patients are tending to occupy beds originally designated for medical emergencies. They need plenty of day-space, and aid in getting around—non-slip polish on the floors, hand-rails in the corridors for example—and occupational therapy to suit their capabilities. They need to be sufficiently active, even if this means nursing staff having to take them out for walks so that they may go to bed tired and content, not needing any sedative drugs to take them through the night. Then they need facilities for care during the inter-current physical illness, including a physiotherapy service to ensure that a few days in bed with a minor ailment does not lead to bronchopneumonia. At Barrow, efforts have been made in this direction. Day space has been contrived, and a special occupational therapy department has been created, but it is all adaptation of already existing facilities, which are being pressed into doing things for which they were not designed, to meet a problem which was perhaps never envisaged. A measure of success has been attained, but the number of old people admitted seems to be rising. Yet, as I hope this survey has illustrated, half of our elderly population could be discharged if only there was somewhere for them to go, and they did not have to fear a return to loneliness once more.

### SUMMARY

A group of elderly patients in a modern psychiatric hospital has been surveyed, and its composition and the problems of this group of patients have been discussed. It was found that half of the patients were not in need of active psychiatric treatment, and could be cared for elsewhere. The practicalities and possibilities of this are discussed.

*Acknowledgements*

I am grateful to Dr. R. E. Hemphill, the Medical Superintendent, for suggesting this to me, and for his critical help in its execution. My thanks are also due to the senior nursing staff and the psychiatric social workers who helped to carry out the survey.

